# Creative Expression of Science through Poetry and Other Media can Enrich Medical and Science Education

**DOI:** 10.3389/fneur.2015.00003

**Published:** 2015-01-22

**Authors:** Sherry-Ann Brown

**Affiliations:** ^1^Department of Medicine, Mayo Clinic, Rochester, MN, USA

**Keywords:** poetic science, poetry, neurology, neuroscience, education, learning, creativity, STEAM

## Introduction

**Figure 1:**
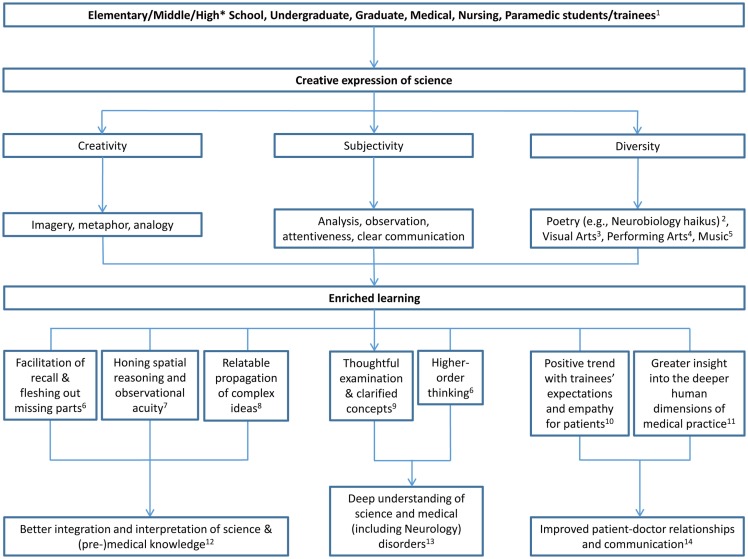
**Creative expression of science enriches learning**. Creative, subjective, and diverse expression of science by elementary and middle school (*and presumably high school, which has not yet been reported) students, as well as undergraduate, graduate, medical, nursing, and paramedic trainees advances their education, based on the structural similarity of using analysis, observation, attentiveness, and clear communication in (i) science and medicine, and (ii) creative expression including poetry, at various stages. This sets up a continuum from elementary school to medical practice. Specifically, the attentive use of imagery, metaphors, and analogy in clearly communicated poetry, music, and visual and performing arts about science and medicine hones skills in analysis and observation. This helps to flesh out missing details and facilitate recall of scientific principles. It also leads to higher-order thinking and greater insight into the human dimensions of medical practice. This results in deeper understanding of science and medicine and improved patient–doctor relationships and communication. ^1^(3–23). ^2^(8, 11–13, 16, 19, 20). ^3^(5, 12, 18). ^4^(3, 4, 6, 7, 9, 10, 15, 17, 21, 23). ^5^(22). ^6^(19). ^7^(18). ^8^(18, 19). ^9^(5). ^10^(16). ^11^(14). ^12^(11, 13, 19, 20). ^13^(11, 12, 19). ^14^(6, 9, 10, 15, 17, 21, 23).

Creative expression of scientific observations and principles through poetry and other media can enrich medical and science education. This debunks the expectation that science and poetry, for example, are mutually exclusive (Figure 1). Instead of coexisting as parallel lanes without interaction, poetry and science can cross over as one merges lanes. Kurtz and Loewenstein suggest that “spontaneous transfer of useful knowledge across domains is a powerful cognitive tool” (1), in this case, the domains of (i) science and medicine, and (ii) creative expression including poetry. Poetry hones critical skills in imagery, metaphor, analogy, analysis, observation, attentiveness, and clear communication. All of these are commonly useful in understanding, problem-solving, and decoding scientific and medical mysteries. Creative science expression facilitates this skills transfer, is not limited to poetry, and can include other media such as visual or performing arts. Active use of metaphors in this way helps learners understand science, and relies on their imagination to deconstruct and construct their perception of science. This serves as a vehicle for processing observations and assumptions, and can enrich education and facilitate learning. Jemison, a doctor, dancer, and astronaut embraces this kind of collaboration and teaches that science and arts are avatars or manifestations of the same human creativity (2).

## Learning through Creative Expression of Science Involves Synergy

The intermingling of creative imagery, metaphor, and analogy with the expression of science can lead to novel outcomes that are more than the sum of its parts (Figure 1). To illustrate, writing scientific thoughts in the form of poems means that ideas cannot exist merely as a milieu in one’s mind. One has to communicate these true ideas in a way that others would understand. With such reasoning, one more deeply examines literature searches, broadening the scope of research findings and expanding the context for interpretation of results, more than would likely occur in the absence of creative expression. Thus, the process of writing good poetry that truly reflects good science leads to a fuller inquiry, interpretation, and appreciation of science. Several examples are provided as evidence for the impact of creative expression on science learners at various stages, from elementary school to graduate and health care education. Incorporating hands-on poetry and artistic expression of science from early stages in education may prime learners for further creative expression integration in advanced science and medical education.

### Undergraduate and graduate education pertinent to neurology and neuroscience

One example involves undergraduate students in Massachusetts, USA and undergraduate and graduate students in New York, USA. Students were instructed to write haikus to learn and accurately convey complex neurological concepts (19). Numerous haikus were written about the symptoms, genetics, and underlying pathology of Alzheimer’s, Huntington’s, and Parkinson’s diseases, among others. Student comments on the exercises’ outcome indicated deep understanding of the neurodegenerative and addiction disorders. Profound learning resulted from internalizing the scientific information, while constructing coherent, impactful, and efficient haikus. Students concisely expressed neurobiological concepts, by emphasizing salient features. Learners were then required to provide written and oral deconstruction of their haikus to walk peers through their evidence-based thought process. The exercises encouraged higher-order thinking to forge a bridge between the arts and sciences and facilitated comprehension and propagation of complex ideas. Writing and sharing haikus helped with recall of the material and fleshing out missing parts. Educators noted that haiku-prose paired responses were the most scientifically accurate and well-written of all the students’ homework assignments.

An unrelated author Chudler compiled “The Little Book of Neuroscience Haiku,” which includes explanations of topics for use in neurology and neuroscience education (24). Using such a model could potentially be a powerful impetus for undergraduate, graduate, medical, and younger learners of neurobiology. Walders supports this concept and suggests that poetry can braid curriculum areas by weaving concepts together (25).

Embracing the diversity of creative science expression, Gurnon et al. suggest that visual arts can help hone creativity, objectivity, perseverance, spatial reasoning, and observational acuity (18). These authors reported on collaboration among faculty and undergraduate students in chemistry and sculpture to produce visual artwork inspired by protein-folding research (18). Their students performed literature searches to learn more about Villin, a calcium-dependent actin-binding protein. Results of the art-science collaboration were presented, using metaphors to help make the science of protein structure and folding more intuitive and meaningful to peer learners. Felton et al. support this form of creative expression and indicate that carefully producing artistic images can lead researchers to thoughtfully examine and clarify concepts behind a scientific topic (5). In this way, metaphoric analysis can help elucidate difficult topics in science.

Taken together, these examples are evidence that creative science expression can enrich undergraduate and graduate education relevant to neurology and neuroscience.

### Medical and nursing education pertinent to neurology

Morris argues that developing writing skills creates better communicators, which equals better doctors (4). Demonstrating this, programs developed for medical residents, with expressive writing focused on difficult patients, cross-cultural medicine, stroke, and caregivers, among others, have led to improved interpersonal relationships and communication (3, 6, 7). Programs for various groups of medical (and premedical) students that enhance observation, interpretation, and imagination skills through creating poetry as well as visual and performing arts, have also improved patient–doctor relationships and communication (6, 9, 10, 15, 17, 21, 23). These studies’ authors concluded that various forms of creative reflection should be encouraged in medical schools and residencies, with extension to practicing health professionals. This is in keeping with Rowe’s analysis of the positive instructive role that writing poetry played in the lives and careers of several physicians and scientists (26). Similar results have been observed for paramedic students, who felt their created works gave great insight into the deeper human dimensions of their practice, inspiring intellectual transformations (14).

Raingruber also wrote poetry about patient experiences in mental health and trained nursing students in this medium (8). She concluded that poems teach students to think holistically and should be more avidly incorporated into education and practice. Webster too incorporated poetry-writing and other creative expression into education of nursing students in psychiatry, with exercises leading to a positive trend in learners’ relationships with patients, expectations, perceptions, understanding, and empathy (16). Foureur et al. also contributed to the training of nurse graduates, by giving them a voice to express the complexity of patient outcomes (27).

These cases are supportive evidence for creative science expression in health professional education.

### Elementary and middle school science education

A number of authors have also used these interdisciplinary methods in elementary and middle school science education. Frazier et al. used science poetry among elementary school students in Virginia, USA (13). In pairs, students created poems about results of their scientific investigations, leading to data-driven consensus about the nature of science. Barbosa et al. also integrated poetry about science among more than one hundred elementary and middle school students in southern Brazil (20). Teachers and students wrote poems about scientific concepts in physics, chemistry, and biology. The interdisciplinary method resulted in stimulating students’ imagination and science knowledge integration. Similarly, Cabrera studied incorporation of science poetry among middle school students in Northern California, USA (11). In the small sample of students, pre- and post-science quiz results showed improvement of up to 13% in overall score. Cabrera concluded that poetry sessions were effective as a tool for science education.

Osborn’s SAW workshops used creative science expression to encourage students’ imagination and exploration of chemical pigment science (12). They helped students learn to correctly identify plant extract pigments by creation of “rainbow tubes” when a variety of acids and alkalis were added to samples. Students’ output reflected the rich contributions of poetry-writing, and working with pigments in class, to their understanding of science. Watts catalogs a number of other settings, in which creative expression of science can or has been used as an innovative tool in science education (28). He considers that school science can be a scientific and literary experience – an esthetic poetic experience.

These examples provide evidence for creative expression of science in enriching elementary and middle school education, which are precursors for medical education.

## Science Expression through Poetry and Other Media is Creative, Subjective, and Diverse

Because science and poetry share creativity, analogy, discipline, knowledge, and imagination in common, Bono recommends convergence between science and literature as interdependent fields (29). This can be captured in creative expression of science (Figure 1).

To demonstrate, the creativity of a scientist draws on imagination, originality, and ingenuity, emerging from a poetic sense of freedom (30). Boxenbaum mentions “creative worrying,” in which the scientist carries around thoughts of his research in the conscious and unconscious realms until a uniquely gratifying association is realized and is communicated to others in the form of new insight. Another way of conceiving this is as if one’s thoughts are a bus driver that stops at several locations, picking up and letting off passenger ideas. The ideas interact with each other in the creativity bus, while being thoughtfully transported to their final destination. An esthetic overlap results that informs and mediates a creative understanding of the science.

In addition to creativity, Uri Alon’s discussion of *How To Choose a Good Scientific Problem* asserts that (choosing questions to study in) science has subjective and emotional aspects (as humans interacting with nature) and that recognizing this can enrich our science and our well-being (31). Anne Osbourn, in her essay *The Poetry of Science*, observes too that scientists like artists (as humans interacting with the world around us) are subjective (32). Accordingly, Raquell Holmes introduces *Improvisational Theater for Computing Scientists*, designed to develop awareness of the creative and subjective capacity of individuals and groups in science education and research (33). In some sense, the message from these and other artist scientists is that subjectivity and creativity can shake hands with each other and nod at science. Science then smiles back, having found deeper meaning and perspective.

The creativity and subjectivity of creative science expression can be encountered in diverse forms. The Smith College Museum of Art displayed Daniel Kelm’s compilation entitled “Poetic Science,” featuring tooled leather and other inventive forms presenting science mixed with poetry and philosophy (34). The Museum of Contemporary Art of Puerto Rico exhibited video, sculpture, and performance from The Wilderness Science and Art Collaboration entitled “Poetic Science” (35). Websites display art, songs, and poems to encourage creative science learning (36, 37). These provide visual and auditory access to creative expression and train science learners to pursue metaphor and imagination. Some K-12 curricula boast methods to encourage such interdisciplinary partnerships in science education. For example, the Waldorf method used widely in various schools formalizes these partnerships (22). The method uses poetry, music, and physical movement to conceptualize mathematics and to help develop spatial and scientific intelligence. Fuglei and others support these partnerships, and also encourage assisting students to use art, pigments, and light to create visual representations of scientific principles (38), to expand the diversity of science expression to enrich learning.

## Collaborative Teams and Resources

Interdisciplinary teams of scientists, poets, and artists (12), and also teachers and physicians, could become the norm. Scientists and poets can become poised to join these teams by accepting Stewart’s proposal of poetry as a means of forging the connection between literacy and science teaching (39). Stewart encourages scientists to consistently incorporate poetry into communicating science. Timpane exhorts scientists to read poetry and poets to read science lifelong (40). Perhaps by doing so, these teams can be effectively incorporated into science education from elementary school through undergraduate, graduate, medical, postgraduate, and continuing education.

Resources are available for developing curricula for creative science expression (41, 42, 43). These may be of particular utility for embracing the arts, including poetry, as a component of science, technology, engineering, arts, and mathematics (STEAM) (2, 44, 45) education.

## Author Contributions

Sherry-Ann Brown conceived, analyzed, designed, drafted, critically revised, approved, and agreed to be accountable for this submitted work.

## Conflict of Interest Statement

The author declares that the research was conducted in the absence of any commercial or financial relationships that could be construed as a potential conflict of interest.
